# Long-term stability of angle-stable versus conventional locked intramedullary nails in distal tibia fractures

**DOI:** 10.1186/1471-2474-14-66

**Published:** 2013-02-20

**Authors:** Dirk Wähnert, Yves Stolarczyk, Konrad L Hoffmeier, Michael J Raschke, Gunther O Hofmann, Thomas Mückley

**Affiliations:** 1 Department of Trauma-, Hand- and Reconstructive Surgery, Friedrich Schiller University Jena, Erlanger Allee 101, Jena, 07747, Germany; 2 Department of Trauma-, Hand- and Reconstructive Surgery, University Hospital Münster, Waldeyerstr. 1, Münster, 48149, Germany; 3 BG-Kliniken Bergmannstrost Halle, Department of Trauma- and Reconstructive Surgery, Merseburger Str. 165, Halle, 06112, Germany

**Keywords:** Distal tibia fractures, Angular stability, Intramedullary nailing, ASLS, Biomechanics, Long-term

## Abstract

**Background:**

In the last years intramedullary nailing has become the treatment of choice for most displaced diaphyseal tibia fractures. In contrast intramedullary nailing of distal tibia fractures is accompanied by problems like decreased biomechanical stability. Nevertheless the indications for intramedullary nailing have been extended to include even more distal fractures. The purpose of this study was to compare long-term mechanical characteristics of angle-stable versus conventional locked intramedullary nails in the treatment of unstable distal tibia fractures. Therefore, the effect of time on the mechanical properties of biodegradable sleeves was assessed.

**Methods:**

8 pairs of fresh, frozen porcine tibiae were used. The expert tibial nail (Synthes) was equipped with either three conventional locking screws (CL) or the angle-stable locking system (AS), consisting of a special ASLS screw and a biodegradable sleeve. Biomechanical testing included torsional and axial loading at different time-points over 12 weeks.

**Results:**

The AS group showed a significantly higher torsional stiffness at all time-points (at least 60%) compared to the CL group (p < 0.001). The neutral zone was at least 5 times higher in the CL group (p < 0.001). The mean axial stiffness was maximum 10% higher (week 6) in the angle-stable locked group compared to the conventional group. There was no significant change of the torsional mechanical characteristics over the 12 weeks in both groups (p > 0.05). For axial stiffness and range of motion significant differences were found in the AS group.

**Conclusions:**

The angle-stable locking system (ASLS) with the biodegradable sleeve provides significantly higher long-term stability. Especially the differences determined under torsional loading in this study may have clinical relevance. The ASLS permits the potential to decrease complications like secondary loss of reduction and mal-/non-union.

## Background

In modern trauma care the treatment of unstable distal tibia fractures is still challenging. In the last years intramedullary nailing has become the treatment of choice for most displaced diaphyseal tibia fractures, because it provides a high mechanical stability and can be performed in a minimally invasive manner [1-7]. Compared to diaphyseal fractures intramedullary nailing of distal tibia fractures is accompanied by problems like decreased biomechanical stability due to the anatomical conditions of the distal tibia [8,9]. The difference in size between the implant diameter and the metaphyseal diameter results in small nail–cortex contact. Additionally the diminished cortical bone support of the distal tibia limits the construct stability [10]. Nevertheless the indications of intramedullary nailing have been extended to include even more distal fractures [9,11]. Consequently, fractures of the distal one third of the tibia treated with intramedullary nailing frequently result in varus, valgus, or torsional deformities and nonunions [12-16].

To improve the construct stability of intramedullary nailed distal tibia fractures, recently, angle-stable interlocking screws encased by a sleeve have been introduced. First using a PEEK (polyetheretherketone) sleeve, the manufacturer completely changed the sleeve material to biodegradable 70:30 poly(L-lactide-co-D,L-lactide). In a previous study our group could already show the significant increase of initial torsional stability due to angle-stable locking [17].

The purpose of this study was to assess the long-term biomechanical fixation characteristics of the angle-stable locking system using the new biodegradable sleeve.

## Methods

The protocol used in this study was based on the publication of Wähnert et al. [17].

### Specimens

**Figure 1:**
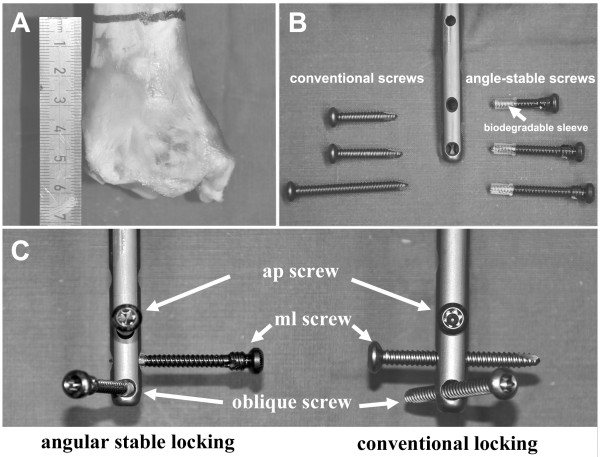
(A) Porcine tibia with osteotomy 5.5 cm proximal to the tibiotalar joint line indicated; (B) cannulated Expert Tibial Nail with according locking screws (conventional – left, angle-stable with sleeves (arrow) preassembled – right); (C) Screw configuration used in this study with three screws for distal locking (ap = antero-posterior, ml = medio-lateral, oblique = antero-lateral to postero-medial).

For this study eight pairs of fresh frozen porcine tibiae (all female, all same age) were used (bought at the Slaughterhouse Jena, Germany). Specimens were frozen, stored at −20°C, and thawed at room temperature 24 hours before potting and mechanical testing. Within each pair, one tibia was randomized to receive angle-stable locking (group I - AS), whereas the contralateral tibia received conventional locking (group II - CL). Before testing, specimens were completely stripped of soft tissues and a transverse osteotomy was performed 5.5 cm proximal to the tibiotalar joint line (Figure 1A).

### Implants

The 8 mm cannulated expert tibial nail (Synthes GmbH, Solothurn, Switzerland) with according locking screws was used (Figure 1B). All nails were cut 20 cm above the distal end. Proximally they were embedded over a length of 5 cm in two component cast resin (RenCast FC 52; Huntsman Advanced Materials, Monthey, Switzerland). An additional hole in antero-posterior direction has been drilled 13 cm proximal to the nail tip to connect the nail to a custom made drill guide jig.

For locking in both groups the three most distal screw holes were used as follows: distal screw from antero-lateral to postero-medial, middle screw from medial to lateral and proximal screw from anterior to posterior (Figure 1C). In group I (AS) special 4 mm ASLS screws were used with the corresponding 4 mm ASLS sleeve. In group II (CL) 4 mm standard locking screws were used. All screws were chosen in appropriate length for a bicortical purchase.

### Instrumentation

**Figure 2:**
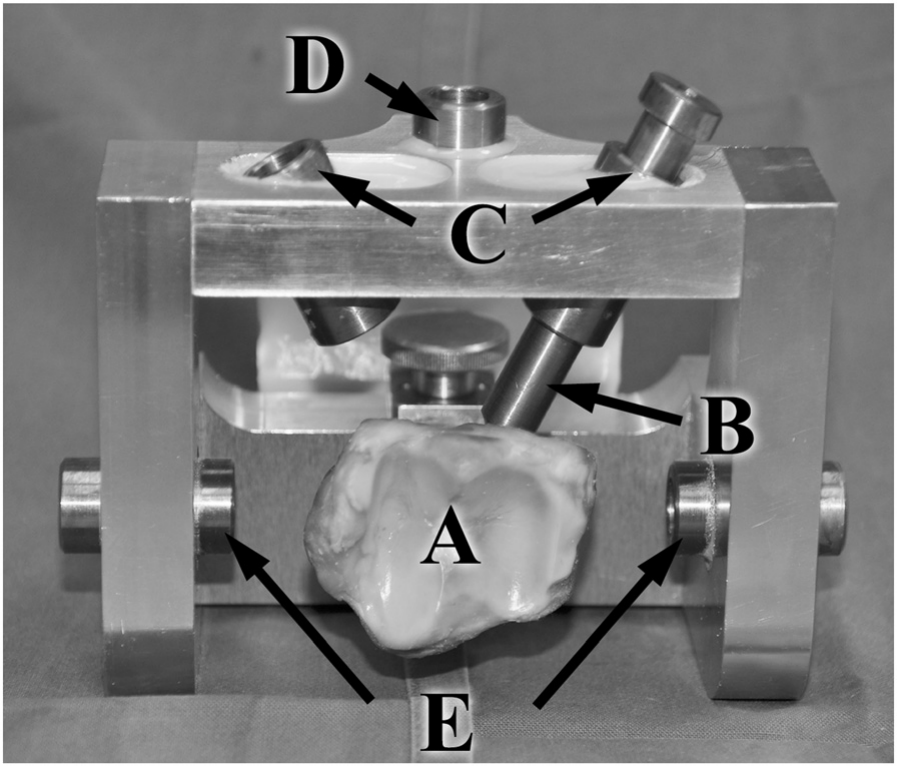
**Distal locking using the custom made locking jig. A**) specimen, **B**) drill sleeve, **C**) oblique drill guides, **D**) anterior-posterior drill guide, **E**) medial drill guides. Following nail insertion the construct was fixed to the jig and standardized drilling and locking could be performed (for left and right tibiae).

The nail was tapped into the unreamed distal tibia part to a distance of 15 mm from the distal articular surface. The position of the nail was checked using an image intensifier. Afterwards the distal locking was performed using a custom made drill guide to ensure standardized distal locking (Figure 2). The locking procedure followed the manufacturer’s surgical technique and all steps were checked with the image intensifier.

Before distal embedding all exposed implant surfaces were covered with modeling compound to prevent direct contact with the two component cast resin. A custom made jig was used for both, distal and proximal embedding to ensure a central nail position. Thus torsional loading without any bending was assured.

### Mechanical testing

**Figure 3:**
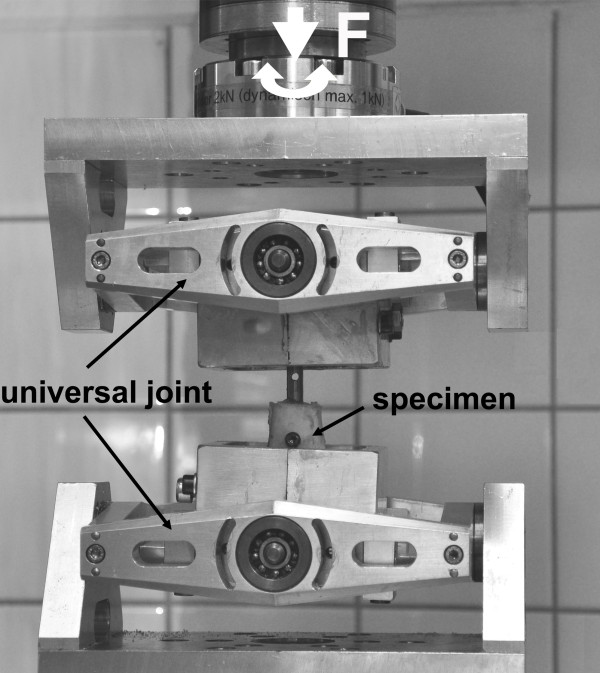
Test setup for axial and torsional testing.

Quasi-static mechanical testing was performed on a servo-hydraulic testing machine (Instron 8874, Instron, High Wycombe, Bucks, United Kingdom) (Figure 3).

Axial and torsional testing was performed subsequently, starting with torsional loading. The specimens were loaded with 5 Nm in external and internal rotation for 10 cycles with a crosshead speed of 1 Nm/s. Followed by axial loading with a preload of 5 N and a crosshead speed of 1 mm/min to a maximum load of 50 N (tension and compression) for 10 cycles. The loads were chosen to ensure deformation in the elastic range only without damaging the bone implant construct. This protocol was performed for all specimens after instrumentation and was subsequently repeated after four, six, eight and twelve weeks.

### Storage

In between the tests the specimens including the potting and the implants underwent immersion in phosphate-buffered saline (PBS). The temperature (37 degrees C) and the pH (7.4) were kept constant throughout the period of immersion [18].

### Data acquisition and evaluation

Time, load, displacement, torsional moment, angle and cycle number were acquired and plotted with use of MAX software (version 9.2; Instron, Canton, Massachusetts). Using the load displacement curves axial and tosional stiffness, range of Motion (ROM) and the torsional neutral zone (NZ) were determined following Wilke et al. [19]. Statistical analyses were performed using SPSS for Windows (Version 16, SPSS Inc., Chicago). After assessing data distribution using the Shapiro-Wilk test, significant differences between the groups were identified and analysed using one-way ANOVA (with Bonferroni correction) statistics. Significance level was set to α = 0.05.

## Results

### Torsional

**Figure 4:**
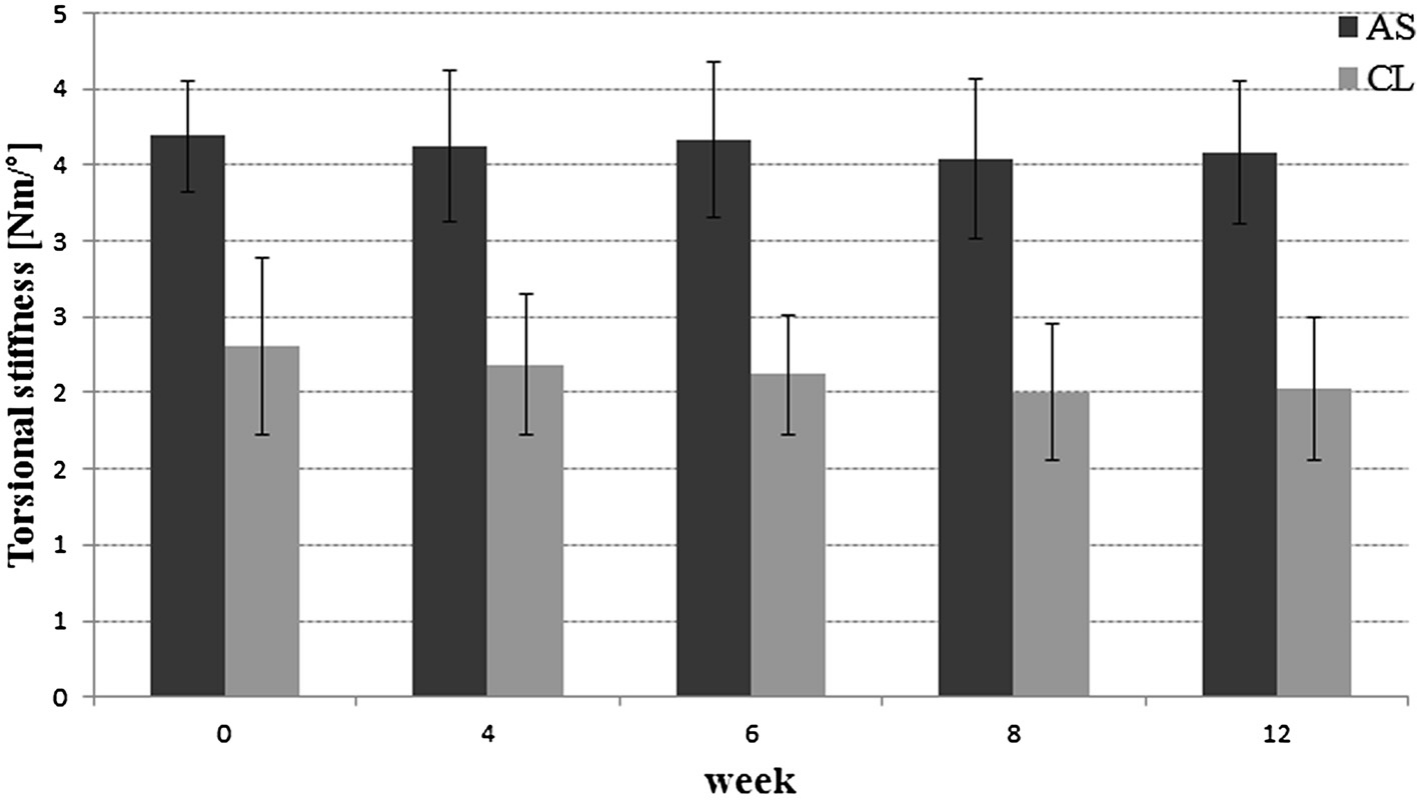
Diagram showing the mean torsional stiffness [Nm/°] for all measured time-points for the angle-stable and conventional constructs with the standard deviation.

For torsional stiffness the angle-stable group also showed significantly higher values at all time-points (p < 0.01). Additionally there was no significant change of the torsional stiffness over time within the groups (AS p > 0.97, CL p > 0.74, Figure 4).

**Figure 5:**
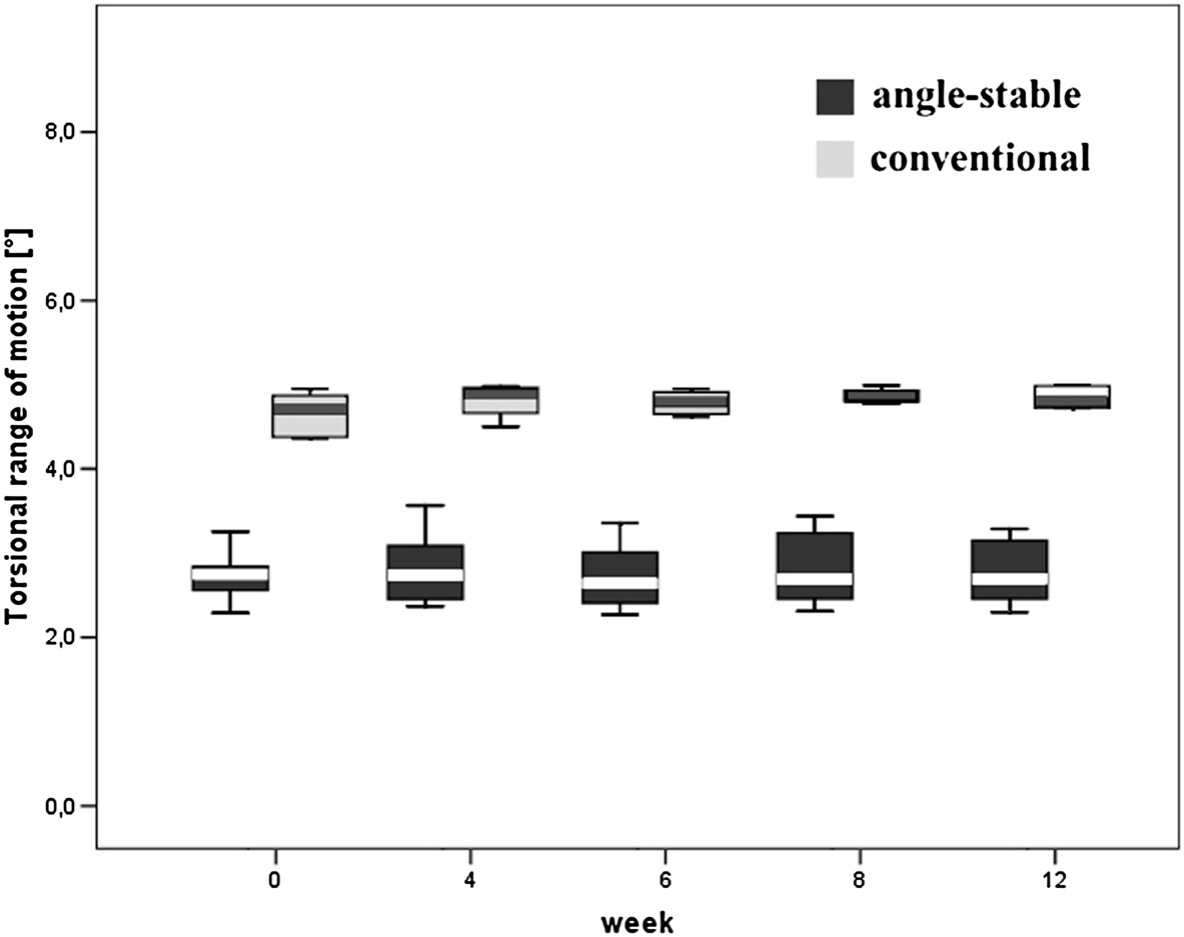
Box plot of the torsional range of motion [°] for the angle-stable and the conventional group.

The torsional ROM was significantly lower for the angle-stable locked group at any time-points compared to the conventional locked specimens (p < 0.01; Figure 5). For the conventional group the ROM was approximately 70% larger compared to the angle-stable. There was no significant change of the ROM comparing the different time-points in both groups (AS p > 0.98, CL p > 0.82).

**Figure 6:**
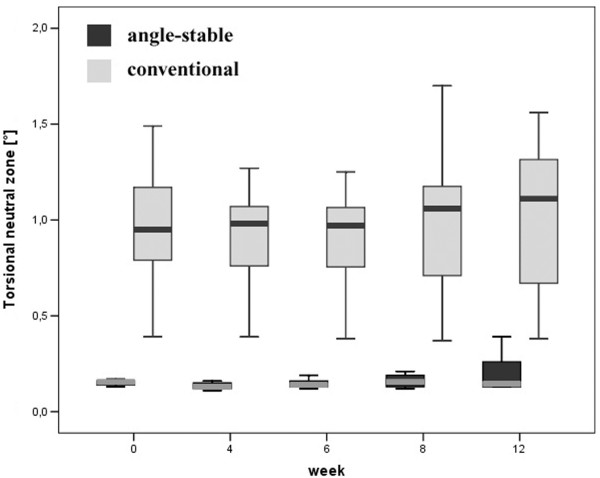
Box plot showing the torsional neutral zone [°] for all measured time-points for the angle-stable and conventional constructs.

The neutral zone was significantly lower for the angle-stable locked construct at all time-points measured (p < 0.01, Figure 6). The conventional locked group had a 5 to 6 times larger neutral zone compared to the angle-stable. For the angle-stable and the conventional group there was no significant change comparing the different time-points (AS p > 0.33, CL p > 0.97).

### Axial

**Figure 7:**
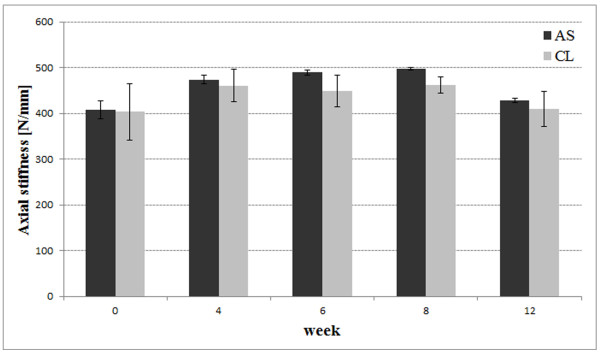
Diagram showing mean axial stiffness [N/mm] with standard deviation for both groups and all time-points.

The mean axial stiffness was maximum 10% higher (week 6) in the angle-stable locked group compared to the conventional group. Statistical this difference was significant for week 6, 8 and 12 (p < 0.02, Figure 7). Comparing week 0 and 4 there was no significance found (p > 0.14). Within the angular-stable group there was a significant increase of stiffness comparing week 0 and 6 (p = 0.024) and week 0 and 8 (p = 0.021). The conventional group showed no significant change comparing the different time-points (p > 0.06).

**Figure 8:**
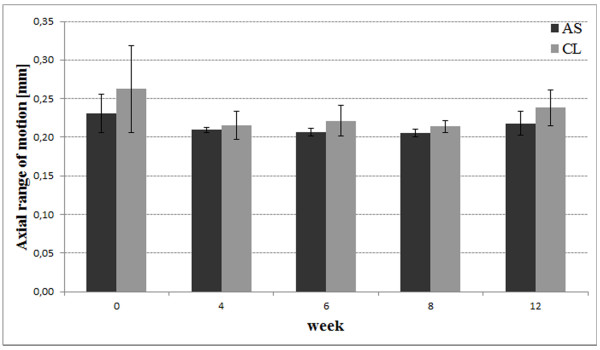
Diagram showing mean axial range of motion with standard deviation for both groups.

The mean axial range of motion was higher for the conventional locked group (maximum 14% in week 0). Statistically significant difference was only found for week 8 (p = 0.019, Figure 8). Within the angular-stable group there was a significant difference comparing week 0 and 6 (p = 0.021) and week 0 and 8 (p = 0.013). The conventional group showed significant differences comparing week 0 and 4 (p = 0.04) and week 0 and 8 (p = 0.03).

## Discussion

This study was performed to investigate the long-term mechanical fixation stability of the angle-stable locking system using the new biodegradable sleeve in the treatment of distal tibia fractures fixed with IM nailing. Angle-stable locking results in a significant increase in torsional fixation stability including a significant reduction of the neutral zone, range of motion and increase of stiffness compared to conventional locking. Additionally the torsional stability showed no significant change over 12 weeks within both groups. For axial loading angle-stable locking had a less powerful impact on stability including stiffness and range of motion. Axial stiffness was significantly higher for the angle-stable group starting from week 6.

Intramedullary nailing of tibia fractures is an accepted and widely used treatment option. In the past few years this method has been used to address even more distal tibia fractures. This extension of the indications goes along with a growing number of reported complications such as delayed healing, nonunion, coronal plane and rotational malalignment/malunion [9,16,20-23].

Various modifications and new developments of implants have been introduced to reduce the complications and make the benefits of intramedullary nailing applicable even in these distal tibia fractures. First of all the number and the sites of the distal locking holes were adapted to the pattern of very distal tibia fractures. In biomechanical and clinical studies shortened tibia nails showed comparable biomechanical stability and good clinical results compared to standard nails [24,25]. Modern intramedullary tibia nails allow distal locking using up to four screws.

One novel approach to the problems of distal tibia fracture management is the angle-stable locking of intramedullary nails with use of a preassembled 70:30 poly(L-lactide-co-D,L-lactide) sleeve on ASLS locking screws. A few previous studies already investigated this technique using a PEEK (polyetheretherketone) sleeve. In summary these studies showed a significantly higher axial stiffness and significantly less fracture gap movement [26] and a significant reduction of the neutral zone in mediolateral bending for the angular-stable locked intramedullary nails [27]. A third study showed the potential of angle-stable locking to maintain fixation stability while reducing the number of locking screws in the treatment of unstable distal tibia fractures [28]. All these studies used the PEEK sleeves. In a former study our group already investigated the primary biomechanical fixation stability of the new biodegradable sleeve. In this study we found the angle-stable locked constructs providing a significantly higher torsional and axial primary stability compared to conventional locking [17]. In the present study we could affirm the advantage of the angle-stable locking especially for torsional fixation stability over a time of 12 weeks. In an in vivo study using tibia midshaft fractures in sheep Epari et al. could show the negative influence of torsional and sheer stresses on fracture healing [29]. Additionally the group of Kaspar et al. found angle-stable locking of intramedullary nails in tibia fractures to result in less fracture gap movement and better radiologic, histomorphometric, biomechanical and clinical fracture healing in sheep [30]. Therefore angle-stable locking of intramedullary nails seemed to be an option to reduce the risk of delayed union and, because of the increased stability, of secondary loss of reduction. Thus, this procedure potentially provides an option to use intramedullary nailing in even distal tibia fractures and osteoporotic fractures.

One concern about the use of biodegradable implants is an inflammatory reaction (seen after medial malleolus fixation). The system is available since 2009 and, to the knowledge of the authors until now no case of inflammation or problems in wound healing have been reported. The sleeve degrades to lactic acid; the degradation speed depends on the location of the sleeve and patient characteristics. The manufacturer (Synthes, Solothurn, Switzerland) guaranteed mechanical characteristics for four weeks. We have chosen the time-points for mechanical measurements from a clinical point of view, the healing process of a tibia fracture treated with an intramedullary nail can take up to 3 month (12 weeks). The huge differences between the sleeve and implants used for medial malleolus fixation are the small amount of material of the sleeve and the total intramedullary and nearly complete intra-nail location of the sleeve. This results in a very small interaction area between human body and biodegradable sleeve.

This study has limitations. First, the use of porcine bone, this material is widely used for biomechanical testing due to its availability [31], for example the knee and spine [32-35]. From these investigations we know that the bone mineral density of the porcine tibia is higher than human [36,37]. For the interpretation of the results we have to take into account that we do not have an osteoporotic bone model. But the advantage of increased stability of the angle-stable locking is due to decreased screw-nail movement and will also be present in osteoporotic bone. Also the number of specimens was small, but it was high enough to show significant differences between the groups. Furthermore, the standard deviation in the AS group was very small. In the CL group it was clearly higher; this may be caused by the locking procedure: if the locking bolts are not placed ideal perpendicular and central in the locking hole they block the bone implant construct and a higher mechanical stability results. Such variables confound the results, but we tried to reduce them e.g. by standardized locking using a custom made drill and locking jig. Second, we used a “hydrolysis chamber” with standardized temperature and pH to simulate physiologic conditions. Although we know this is a model, it is a well-established way to simulate the in-vivo absorption (hydrolysis) of polylactid-polymers [38-42]. Furthermore, loading conditions have been chosen to allow subsequent torsional and axial loading over 12 weeks without causing plastic deformation due to testing. Thus biomechanical loading in this study does not represent physiologic conditions. Additional biomechanical studies investigating the biodegradable sleeve under physiologic loading have to be performed.

Clinical studies will be required to investigate the utility of the technique in the management of these difficult to treat distal tibia fractures and to show the benefit in patient care.

## Conclusion

The angle-stable locking system (ASLS) using a special screw and sleeve locking for intramedullary nails provides significantly higher long-term fixation stability. Especially in torsional loading the differences determined in this biomechanical study may have clinical relevance. We also found differences between the groups for the axial stability, but we think the magnitude is not that relevant for clinical application. From the mechanical point of view this system has the potential to decrease complications like secondary loss of reduction and mal-/non-union. Clinical studies have to confirm these results.

## Competing interest

All authors disclose any financial and personal relationships with other people or organisations that could inappropriately influence (bias) this work. The material was kindly supplied by the manufacturer Synthes.

## Authors’ contributions

DW was involved in planning the study, assisted during the tests, did the data analysis and wrote the manuscript. YS and KH did the instrumentation, performed the tests and collected the data. Additionally KH was involved in the statistical analysis. MR was involved in writing the manuscript and data analysis. GH and TM were involved in planning the study and writing/revising the manuscript and provide supervision. All authors read and approved the final manuscript.

## Pre-publication history

The pre-publication history for this paper can be accessed here:

http://www.biomedcentral.com/1471-2474/14/66/prepub
